# Inhibition of clathrin by pitstop 2 activates the spindle assembly checkpoint and induces cell death in dividing HeLa cancer cells

**DOI:** 10.1186/1476-4598-12-4

**Published:** 2013-01-17

**Authors:** Charlotte M Smith, Volker Haucke, Adam McCluskey, Phillip J Robinson, Megan Chircop

**Affiliations:** 1 Children's Medical Research Institute, The University of Sydney, 214 Hawkesbury Road, Westmead, NSW, 2145, Australia; 2 Institute of Chemistry and Biochemistry & Neurocure Cluster of Excellence, Freie Universität Berlin, Berlin, 14195, Germany; 3 Leibniz-Institut für Molekulare Pharmakologie (FMP), Berlin-Buch, Germany; 4 Chemistry, School of Environmental & Life Sciences, The University of Newcastle, Callaghan, NSW, 2308, Australia; 5 Children’s Medical Research Institute, Locked Bag 23, Wentworthville, NSW, 2145, Australia

**Keywords:** Clathrin, Pitstop, Spindle assembly checkpoint, Metaphase, Cell death, Cancer

## Abstract

**Background:**

During metaphase clathrin stabilises the mitotic spindle kinetochore(K)-fibres. Many anti-mitotic compounds target microtubule dynamics. Pitstop 2™ is the first small molecule inhibitor of clathrin terminal domain and inhibits clathrin-mediated endocytosis. We investigated its effects on a second function for clathrin in mitosis.

**Results:**

Pitstop 2 did not impair clathrin recruitment to the spindle but disrupted its function once stationed there. Pitstop 2 trapped HeLa cells in metaphase through loss of mitotic spindle integrity and activation of the spindle assembly checkpoint, phenocopying clathrin depletion and aurora A kinase inhibition.

**Conclusions:**

Pitstop 2 is therefore a new tool for investigating clathrin spindle dynamics. Pitstop 2 reduced viability in dividing HeLa cells, without affecting dividing non-cancerous NIH3T3 cells, suggesting that clathrin is a possible novel anti-mitotic drug target.

## Background

Cell division (mitosis) results in equal segregation of duplicated chromosomes into two independent daughter cells. Premature chromosome segregation (metaphase-anaphase transition) results in aneuploidy, a hallmark of many human cancers [1]. This adverse situation is avoided by activation of the spindle assembly checkpoint (SAC). This is a signalling pathway consisting of a number of protein complexes that monitor proper mitotic spindle assembly, delaying anaphase onset until all chromosomes are stably attached to kinetochores (KTs) [2]. KTs are a large protein assembly around the centromere of chromosomes that mediates the attachment of chromosomes to the spindle microtubules (MTs) so they can complete segregation. Both SAC activators and inhibitors are well-known targets for several chemotherapeutic agents. Drugs that target MTs such as the taxanes and vinca alkaloids are extensively used for cancer treatment [3]. They stop APC/C-mediated proteolysis and block anaphase onset in a SAC-dependent manner. Despite success in the clinic, drug resistance and toxicity have limited their effectiveness [3]. Interference with KT assembly, impairment of MT motors (e.g. dynein) and interference with MT dynamics also activate the SAC [4]. Thus, a new class of chemotherapeutics are being developed that specifically target key mitotic proteins to either activate or inhibit the SAC, such as aurora A kinase and kinesin spindle protein, respectively [3]. These targeted inhibitors prevent proliferation of most tumour cells *in vitro* and reduce tumour volume *in vivo* by inhibiting growth and/or triggering cell death following SAC activation/ inhibition [3,4]. Many are in cancer clinical trials, such as the aurora A protein kinase inhibitor MLN8054 [5]. They are expected to have a more favourable therapeutic window than current chemotherapeutic agents [3], as they would spare non-dividing cells. The anti-cancer efficacy of these mitotic inhibitors is dependent on their ability to induce apoptosis following mitotic insult. However, they do not always result in cell death [6]. Thus, there is scope for identification of new anti-mitotic targets and the development of new anti-cancer compounds with greater efficacy.

Clathrin is a protein complex of three identical 190 kDa clathrin heavy chains (CHCs) arranged in a trimer (called a triskelion) of three “legs” connected by their C-termini at a central vertex [7,8]. A globular N-terminal β-propeller domain (TD) is found at the end of each clathrin leg (i.e. at the N-terminus of the protein sequence). Clathrin can interact with multiple adapter proteins like amphiphysin via its TD [9]. Clathrin is best known for its roles in endocytosis and TGN/ endo-lysosmal sorting, however, in recent years it has been assigned another non-trafficking function in mitosis. For clathrin-mediated endocytosis (CME), clathrin cycles between the cytoplasmic triskelion and a polymerised coat on vesicles or membranes. During mitosis, clathrin localizes to the mitotic spindle [10-12] where it is involved in organizing and stabilizing spindle MTs [11-13]. It dissociates from MTs during telophase, as the Golgi reforms to participate in its reassembly [14]. The role of clathrin at the mitotic spindle is dependent on both its TD [11] and ability to trimerise as well as its interaction with TACC3 (transforming acidic coiled-coil-containing protein 3) [13]. Aurora A kinase phosphorylates and localises TACC3 to the spindle [15,16]. Phospho-TACC3 recruits clathrin and ch-TOG to the spindle MTs [13] where they bridge together two or three kinetochore fibres (K-fibres) to aid chromosome congression [11] with TACC3 directly interacting with MTs [17,18]. Depletion of clathrin by siRNA causes defective chromosome congression to the metaphase plate and persistent SAC activation [11,19-21]. This is analogous to the effect of aurora A inhibitors which are also SAC activators [22-26]. Aurora A inhibitors also block clathrin recruitment to the spindle by blocking TACC3 recruitment [27]. Thus, it is possible that SAC activation and the anti-cancer properties of aurora A inhibitors may be partly due to blocking clathrin function at the mitotic spindle.

Clathrin requires its TD to associate with the mitotic spindle [11], although the protein(s) mediating its recruitment remains unclear. Preventing this interaction leads to defective congression of chromosomes to the metaphase plate and persistent activation of the SAC. We have recently developed the first small molecule inhibitors of clathrin, pitstop 1 and pitstop 2, which target the TD [28]. These two chemically unrelated small molecules inhibit the association of CHC-TD with clathrin box motif-containing endocytic proteins such as amphiphysin and AP180/CALM, with no affect on three other protein-protein interactions or on dynamin GTPase activity, demonstrating their relative specificity. Pitstop 2 is a potent inhibitor of transferrin (Tfn) uptake in cells and is reversible, with CME being fully restored after a 30 min drug washout, while pitstop 1 is not readily cell permeable. Here, we investigated the effect of pitstop 2 on mitosis to determine if its ability to block the TD function in CME is also true for its second function in the mitotic spindle. We assessed if it possesses anti-mitotic and anti-cancer properties analogous to other SAC activating compounds. We report that pitstop 2 induces mitotic phenotypes consistent with inhibition of clathrin and is anti-proliferative and cytotoxic in a dividing cancer cell, but not a dividing non-cancer cell. Our findings provide strong support to the specificity of pitstop 2 in targeting the TD and indicate that it is a new member of the SAC activating class of anti-mitotic compounds. The use of pitstop 2 has revealed that clathrin has the potential to be a new therapeutic target.

## Methods

### Clathrin, dynamin and aurora kinase inhibitors

Dynole 34–2 and pitstop 2 (in house synthesis) were prepared as 30 mM stock solutions in 100% DMSO and stored at −20°C. Pitstop 2 was purified to >98% purity (HPLC analysis at 254 nm) and NMR 400 MHz H spectrum in DMSO shows <1% of any other impurity; NMR 100 MHz C spectrum in DMSO shows <1% of any other impurity; TLC analysis using a solvent ratio of 9:1 DCM/MeOH shows a single spot visualised by UV. MLN8237 (Life Research Pty Ltd) was prepared as a 3 mM stock in 100% DMSO and stored at −20°C. Drugs were diluted directly into RPMI 1640 medium supplemented with 10% foetal bovine serum (FBS) and 5% penicillin/streptomycin (P/S). Pitstop 1, Pitstop 2, and Pitstop trademarks of Freie Universität Berlin, Children’s Medical Research Institute and Newcastle Innovation Ltd; Dynole 34-2, Dynole, Dyngo–4a and Dyngo are trademarks of Children’s Medical Research Institute and Newcastle Innovation Ltd are they are available from Abcam Biochemicals® (Cambridge, UK).

### Cell culture, cell synchronization and drug treatment

HeLa cells were maintained in RPMI 1640 medium supplemented with 10% FBS and 5% P/S and grown at 37°C in a humidified 5% CO_2_ atmosphere. For mitotic synchronization, cells were synchronized at the G_2_/M boundary by treatment with the selective cdk1 small-molecule inhibitor, RO–3306 (9 μM) for 18 h as previously described [29,30]. Cells were allowed to progress through mitosis following RO-3306 wash-out. Immediately following RO-3306 removal (i.e. release from the G_2_/M boundary), cells were treated with pitstop 2, dynole 34–2, MLN8237, drug-free medium or 0.1% DMSO vehicle.

### siRNA oligonucleotides

All specific siRNA oligonucleotides were synthesised by Invitrogen. Sequences of the siRNAs targeting the indicated proteins are as follows: clathrin heavy chain: 5’-GCAATGAGCTGTTTGAAGA-3’ and epsin 5’-GGAAGACGCCGGAGTCATT -3’ [12,31]. Luc siRNA, 5’-CGUACGCGGAAUACUUCGATT -3’, was used as a negative control.

### Cell transfection

For siRNA analysis, cells were seeded at 50-60% confluence (6–well plate-1 × 10^5^ cells per well; 12–well plate-0.2 × 10^5^ cells per well) and transfected with 1000 pmol of siRNA (per 10 cm dish for immunoblotting), or with 200 pmol (per well of a 6–well plate for immunofluorescence microscopy experiments) or 100 pmol (per well of a 12-well plate for immunofluorescence microscopy experiments), using Lipofectamine 2000 (Invitrogen) according to the manufacturer’s instructions.

### Immunoblotting

Cell lysates were prepared as described previously [32]. In brief, cells were collected by centrifugation, washed with PBS, then resuspended in ice-cold lysis buffer (20 mM Tris-HCL (pH 7.4), 150 mM NaCl, 1 mM EDTA, 1 mM EGTA, 1% Triton X–100 and EDTA-free Complete protease inhibitor cocktail (Roche)) for 30 mins. The supernatant (cell lysate) was collected following centrifugation at 13,000 rpm for 30 min at 4°C. Cell lysates (50 μg) were fractionated by SDS-PAGE for immunoblot analysis. Antibodies targeting the following proteins were used: cleaved PARP (Cat No. 9542S, Cell Signalling) and actin (A3854, Sigma). Antibody bound to the indicated protein was detected by incubation with a horseradish peroxidase-conjugated secondary antibody (Sigma). Blotted proteins were visualised using the ECL detection system (Pierce).

### Immunofluorescence microscopy

Cells were fixed in ice-cold 100% methanol and immunostaining was carried out as described previously [33] using the following antibodies: anti-γ-tubulin (GTU88; Sigma-Aldrich), and anti-α-tubulin (clone DM1A, Sigma-Aldrich), anti–Clathrin heavy chain (BD Biosciences), anti-Centrin 2 (a gift from Eric Nigg) anti-tubulin (Pan, Cytoskeleton) anti-MAD2 (Covance), anti-phospho-BubR1^S676^ (a gift from Sabine Elowe [34]), anti-HURP (Abcam). Cells were viewed and scored with a fluorescence microscope (Olympus BX51). Fluorescent images were captured and processed using an Olympus IX80 inverted microscope using 60× or 100× oil immersion lenses and Metamorph software. Images were deconvolved using AutoQuant X2 (AutoQuant Imaging, Watervliet, NY). Spindle width and cell width was determined using the line measurement tool in Metamorph software.

### MT regrowth after cold depolymerization

Asynchronously growing or metaphase synchronized cells were placed on ice for 30 min to depolymerize all MTs. Cells were then incubated at 37°C for 1, 5 or 30 min to allow the MTs to regrow. Cells were then fixed with ice cold methanol, stained for α-tubulin and γ-tubulin and fluorescent images were obtained as described above. The extent of spindle MT regrowth was assessed by determining the length of the longest microtubule using deconvolved images and the line measurement tool in Metamorph software.

### Lactate dehydrogenase (LDH) cytotoxicity assay

Cytotoxicity was assayed by determination of lactate dehydrogenase (LDH) activity. HeLa cells (4000 cells per well) were seeded in 96 well plates. Asynchronously growing and G2/M-synchronized cells were treated in triplicate with pitstop 2, dynole 34–2 or MLN8237 at the indicated concentration for 20. The supernatant (50 μl) was added to 150 μl of LDH assay reagent (Sigma-Aldrich) and the reaction was allowed to developed for 20 min. Absorbance was measured at 490 nm and 690 nm (plate background absorbance). Values were normalised to drug/media background value and toxicity was calculated as a percentage of a control cell lysed with 20% Triton–X–100.

### Trypan blue exclusion assay

HeLa were seeded in 6-well plates (0.5 × 10^5^ cells per well). On day 0 (24 h after seeding), cells in duplicate were synchronized at the G_2_/M boundary by treatment with RO–3306. Immediately upon mitotic entry (RO–3306 release), cells were treated in the presence or absence of pitstop 2 or dynole 34–2, at a concentration of 1, 3, 10, 30, or 100 μM or with MLN8237 at 0.03, 0.3, or 1 μM. After 24 h, the total cell number and viability were measured using a Vi-CELL XR cell viability analyser as previously described [30]. Where indicated, cells were treated a second time with the same compound for an additional 24 h and then analysed.

NIH3T3 cells were seeded in 6-well plates (0.5 × 10^5^ cells per well). On day 0 (12–16 h after seeding), the media was replaced with low serum DMEM (0.5% FCS) to synchronize the cells at the G_1_/S boundary. After 24 h, the low serum media was replaced with DMEM containing 10% FCS to allow cells to synchronously enter the cell cycle. After 10 h, when cells had begun to enter mitosis, cells were treated and scored for viability as described above for HeLa cells.

### Endocytosis assay

Quantitative high-throughput receptor-mediated endocytosis (RME) assays were performed by an automated process using Texas Red-Transferrin (T×R-Tfn) as previously described [35,36] in HeLa cells pre-treated with increasing concentrations of pitstop 2 and dynole 34–2 for 30 min. In brief, HeLa cells were grown in fibronectin-coated (5 *μ*g/mL) 96–well plates. The cells were serum-starved overnight (16 h) in DMEM minus FCS then incubated with dynole 34-2, pitstop2 or vehicle for 30 min prior to addition of 4 *μ*g/mL of Tf-×R for 8 min at 37°C. Cell surface-bound Tfn was removed by an ice-cold acid wash (0.2 M acetic acid + 0.5 M NaCl, pH 2.8) for 10 min then rinsed with ice-cold PBS for 5 min. Cells were immediately fixed with 4% PFA for 10 min at 37°C. Nuclei were stained using DAPI. Quantitative analysis of the inhibition of T×R-Tfn endocytosis in HeLa cells was performed on large numbers of cells by an automated acquisition and analysis system (Image Xpress Micro, Molecular Devices, Sunnyvale, CA). Nine images were collected from each well, averaging 40–50 cells per image. Average integrated intensity of the Tfn signal/cell was calculated using the IXM software and expressed as a percentage of DMSO-vehicle treated control cells. IC_50_ values were calculated using Prism 5 (GraphPad Software Inc.) and data are expressed as mean ± 95% confidence intervals (CI) for triplicates and ~1200 cells.

## Results

### Pitstop 2 inhibits the mitotic spindle

**Figure 1:**
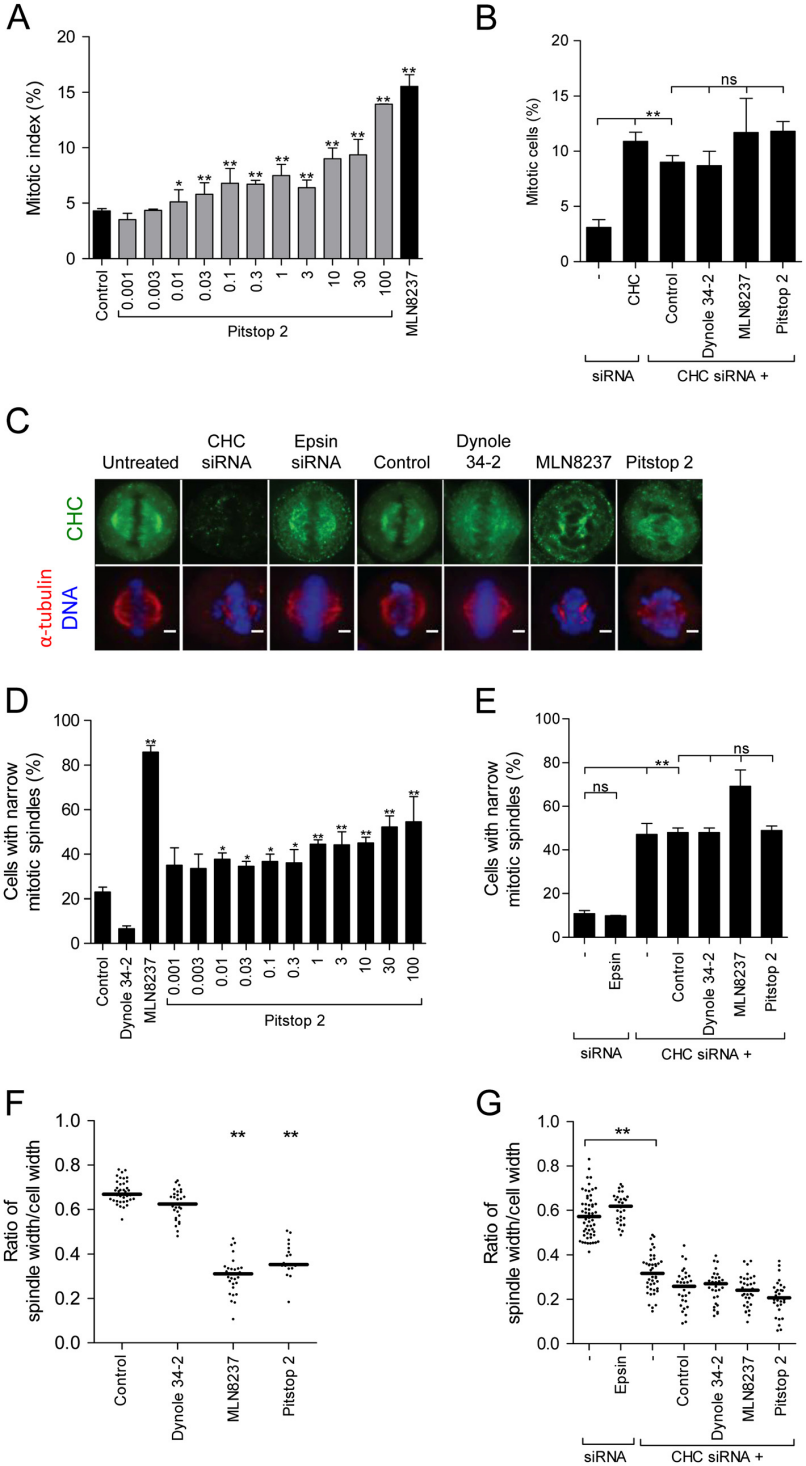
**Pitstop 2 disrupts the mitotic spindle and impairs mitotic progression. A-B**, Asynchronously growing HeLa cells were treated with the indicated concentration of pitstop 2, 0.1% DMSO vehicle, or 0.3 μM MLN8237 for 6 h **(A)**. Asynchronously growing CHC-depleted cells were treated with pitstop 2 (30 μM), dynole 34-2 (30 μM), MLN8237 (0.3 μM) or DMSO vehicle (0.1%) for 6 h **(B)**. Graphs show the percentage of cells in metaphase (mitotic index, mean ± S.E.M. from two independent experiments, n≥300 per sample). **(C)** Representative microscopy images of G_2_/M-synchronized HeLa cells after 30 min treatment with pitstop 2 (30 μM), dynole 34-2 (30 μM), MLN8237 (0.3 μM) or DMSO vehicle (0.1%). Images of untreated, CHC-depleted and epsin-depleted G_2_/M-synchronized cells are also shown. Alpha-tubulin (red); CHC (green); DNA (blue).**D-E**, Graphs (mean ± S.E.M. of two independent experiments; n≥30 per sample) show the percentage of metaphase cells with a narrow mitotic spindle in **(D)** cells described in C as well as in **(E)** untreated, CHC-depleted, epsin-depleted G_2_/M-synchronized and in CHC-depleted cells treated with pitstop 2 (30 μM), dynole 34-2 (30 μM), MLN8237 (0.3 μM), or DMSO vehicle (0.1%) for 30 min following G_2_/M synchronization. **F-G**, Dot blots show the ratio of the width of the mitotic spindle/total cell of metaphase cells quantified in (D) and (E). Pitstop 2 assessed at 30 μM. Each dot represents one cell. Solid black line represents median (n≥2, 30 cells per sample). Statistical significance determined by Student’s *t*-test (* p < 0.05, ** p < 0.01).

CHC localises to the mitotic spindle and CHC depletion results in an increase in mitotic index (i.e. accumulation of cells at metaphase) with metaphase cells having a collapsed mitotic spindle with a broad metaphase plate [11]. The mitotic roles for clathrin have been identified through the use of molecular tools, such as siRNA and overexpression constructs, that take days to exert their effects. Therefore, we aimed to confirm a role for clathrin at the mitotic spindle by using the small molecule clathrin inhibitor, pitstop 2, which penetrates cells and exerts it biological effect within minutes. To determine whether pitstop 2 (key compound structures are shown in Additional file 1: Figure S1) might mimic the effect of CHC knockdown, HeLa cells were acutely treated for 6 h with pitstop 2. The mitotic index increased significantly (Figure 1A), suggesting that it activates the SAC. The effect on mitotic index was concentration-dependent, being significant at concentrations as low as 0.01 μM. At 100 μM, 14.0 ± 0.02% of the cells were in metaphase, compared to 4.4 ± 0.6% of control cells (treated with the vehicle, DMSO). A similar effect was observed after treatment with the Aurora A protein kinase inhibitor, MLN8237, (15.5 ± 2.3%; Figure 1A), which is a SAC activator with an unrelated chemical scaffold. To ask whether the pitstop-mediated phenotype was due to targeting the TD of CHC, we tested its ability to alter the mitotic index of CHC-depleted cells using siRNA, which depleted this protein by >90% as we have previously characterised (Smith, 2012 21118 /id; Additional file 2: Figure S2). As previously reported [11], the mitotic index of CHC-depleted cells was significantly elevated (10.9 ± 0.8% vs 3.1 ± 0.7% for untreated control cells; Figure 1B). Treatment of CHC-depleted cells with pitstop 2 did not have any additional effects, nor did the dynamin inhibitor, dynole 34-2, which exclusively disrupts cytokinesis [37]. MLN8237 had no further effects (Figure 1B), consistent with the idea that clathrin acts downstream of Aurora A. Thus pitstop 2 disrupts HeLa cells from completing metaphase, consistent with inhibition of the TD of CHC and SAC activation.

**Figure 2:**
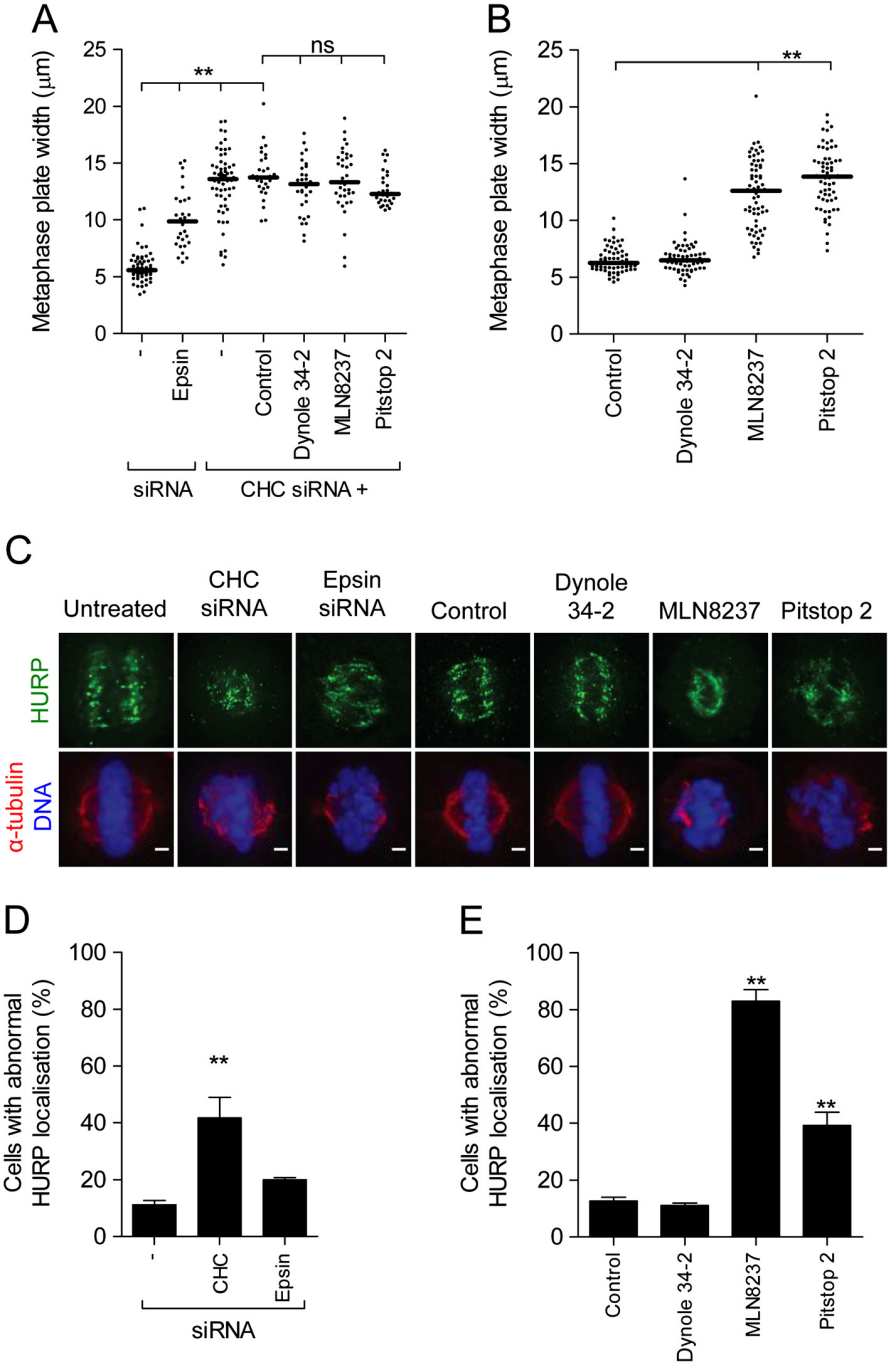
**Pitstop 2 disrupts chromosome congression and K-fibre organization during metaphase. A-B**, Metaphase synchronized HeLa cells were treated with the indicated siRNA either alone or in combination with pitstop 2 (30 μM), dynole 34-2 (30 μM), MLN8237 (0.3 μM), or DMSO (0.1%) **(A)** or with the indicated drug alone **(B)**. The graph shows the width of the metaphase plate in each experimental condition, where each dot represents a cell (n≥2 30 cells were scored per sample). Median is indicated by the solid black line. Statistical significance was determined by a Student’s *t*-test (* p < 0.05, ** p < 0.01). **C-E**, Metaphase synchronized HeLa cells treated with the indicated siRNA or drugs were stained for α-tubulin (red), HURP (green), and DNA (DAPI, blue). Representative immunofluorescence microscopy images are shown in **(C)**. The graphs (mean ± S.E.M.; n≥30 per sample) show the percentage of siRNA-treated **(D)** and drug-treated **(E)** cells with abnormal HURP localisation. Statistical significance was determined using a Student’s *t*-test (* p < 0.05, ** p < 0.01).

We next analysed mitotic spindle phenotypes induced by pitstop 2 in more detail (Additional file 3: Figure S3). The width of the mitotic spindle at the cell equator was reduced in 54.6 ± 11.3% of metaphase cells treated with pitstop 2 (100 μM) as revealed by immunofluorescence microscopy analysis (Figure 1C and D). These spindles appeared to contain less MT fibres (Figure 1C). The narrow or collapsed mitotic spindle phenotype was observed in a significant number of metaphase cells at pitstop 2 concentrations as low as 0.01 μM. An analogous phenotype was observed following exposure to MLN8237 (Figure 1C and D), as previously reported [38]. Metaphase cells depleted of CHC also had a collapsed mitotic spindle (47.1 ± 5.0% vs 10.9 ± 1.4% for control cells; Figure 1C and E). In contrast, inhibition of dynamin with dynole 34-2 (30 μM, Figure 1C and D) or depletion by siRNA of another endocytic protein, epsin, that also has a role in metaphase (Additional file 2: Figure S2) [39], did not collapse the spindle (Figure 1C and E). The extent of mitotic spindle collapse was quantified as a ratio of the spindle width:total cell width at the cell equator and was unaffected by dynole 34–2 or epsin siRNA (Figure 1F and G). Pitstop 2, MLN8237 and CHC siRNA all reduced the ratio from 6.5 to <0.4 (Figure 1F and G). Pitstop 2 did not further reduce this ratio after CHC siRNA treatment (Figure 1G), again supporting that pitstop action is mediated via clathrin. As previously reported [11], in cells depleted of CHC there is a broader metaphase plate (13.6 μm vs 5.6 μm in control cells; Figure 2A) indicative of clathrins’ role in chromosome congression. Pitstop 2 (13.9 μm) and MLN8237 (12.6 μm produced an analogous phenotype, Figure 2B) and again pitstop 2 had no additional effect in CHC-depleted cells (Figure 2A).

We next asked whether the reduced number of mitotic spindle fibres observed in pitstop 2–treated metaphase cells was due to disruption of K-fibres. CHC stabilizes the mitotic spindle by specifically bridging K-fibres [11,17,18]. As would be predicted from this, we found that metaphase cells depleted of CHC also have abnormal staining of the K-fibre marker, hepatoma upregulated protein (HURP; Figure 2C and D). Pitstop disrupted HURP staining in 39.2 ± 4.6% of metaphase cells (Figure 2C and E). MLN8237 and epsin depletion also disrupted HURP staining (Figure 2C-E), while dynole 34–2 had no effect (Figure 2C and E). Therefore pitstop 2 disrupts mitotic spindle organisation by disrupting K-fibres, resulting in impaired chromosome congression to the metaphase plate.

The mitotic effects induced by pitstop 2 cannot be attributed to a block in endocytosis as both pitstop 2 and dynole 34–2 blocked uptake of transferrin in a dose-dependent manner (Additional file 4: Figure S4), as previously reported [28,35]. However only pitstop 2 disrupted the mitotic spindle and chromosome alignment (Figure 1 and 2). This is consistent with the mitotic role of CHC being known to be independent of its endocytic function [11].

### Pitstop 2 induces multipolar spindles and loss of centriole cohesion

**Figure 3:**
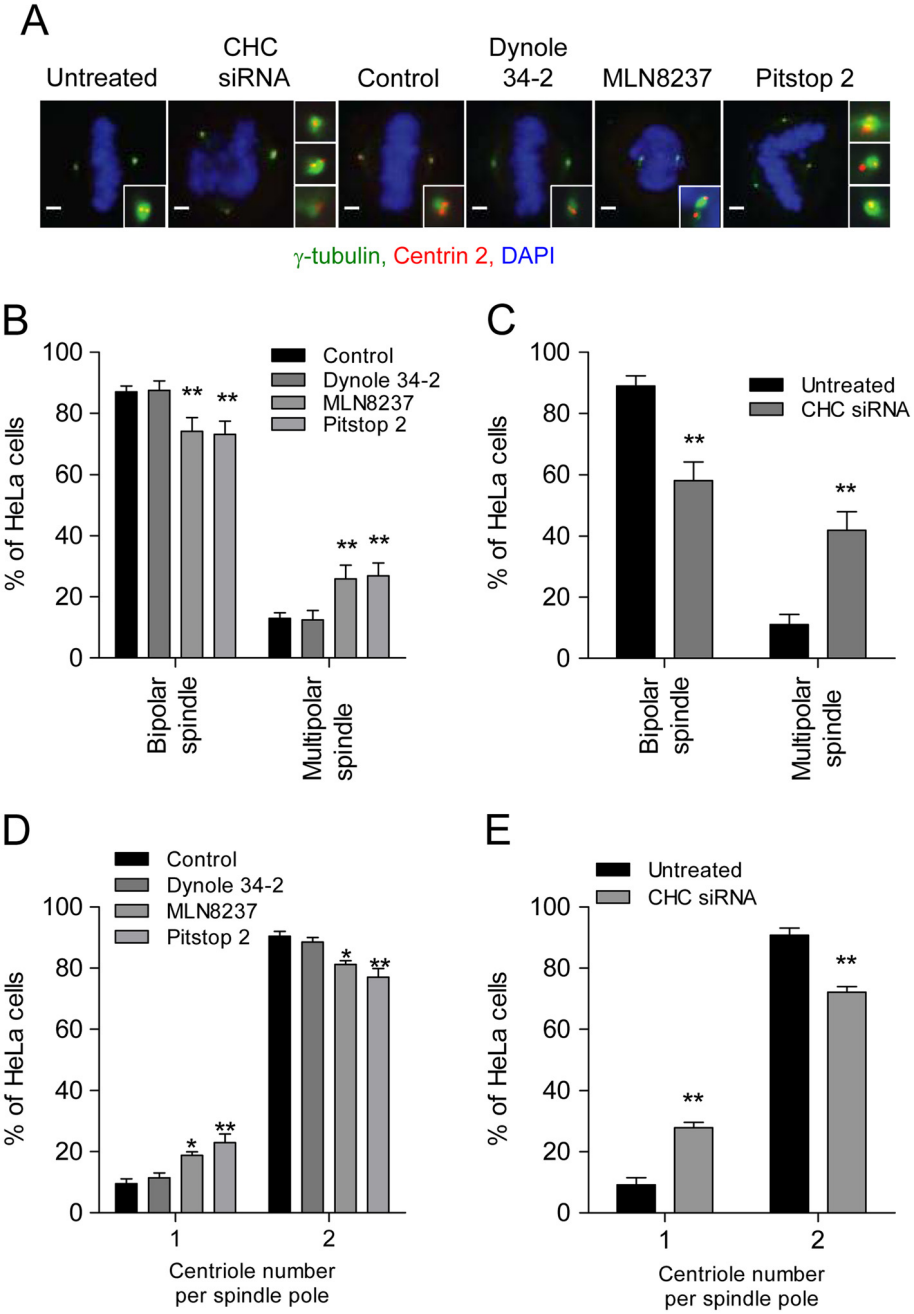
**Pitstop 2 induces multipolar spindle formation and loss of centriole cohesion. A**, Representative immunofluorescence microscopy images of metaphase synchronized CHC-depleted HeLa cells or HeLa treated with pitstop 2 (30 μM), dynole 34-2 (30 μM), MLN8237 (0.3 μM) or DMSO vehicle (0.1%) showing the centrosomes (centrin 2, red) surrounded by the PCM (γ-tubulin, green). DNA shown in blue. Inset shows a zoomed in image of the centrosomes. **B-E**, HeLa cells treated with the indicated drugs **(B **and **D**) or siRNA (**C** and **E**) described in (**A**) were scored for the presence of multipolar spindles (**B** and **C**) and the poles of these spindles were scored for the number of centrioles per PCM (**D** and **E**). The graphs (mean ± S.E.M. of two independent experiments; n≥30 per sample) show the percentage of cells with these characteristics. The x-axis in **D** and **E** represent the number of centrioles per spindle pole in each metaphase cell. Statistical significance was determined by a Student’s*t*-test (* p < 0.05, ** p < 0.01).

Depletion of CHC increases the frequency of multipolar spindles in 32.0 ± 3.6% of cells (Figure 3A and C), as previously reported [40]. Pitstop 2 also induced multipolar spindles in 26.4 ± 3.4% of metaphase cells vs 6.4 ± 3.1% or 9.6 ± 2.5% for untreated or vehicle-treated cells (Figure 3A and B). The spindle pole (or centrosomes) of a metaphase cell is the site of microtubule nucleation and is composed of a mother and daughter centriole, surrounded by pericentriolar matrix (PCM) [41]. Pitstop 2 treatment reduced the number of centrioles to one in a significant number of spindle poles (26.8 ± 1.0% vs 6.0 ± 2.1% of control cells; Figure 3A and D). This action was phenocopied in cells depleted of CHC (24.2 ± 3.9%; Figure 3A and E), suggesting that CHC contributes to centriole cohesion. MLN8237 treatment caused an analogous phenotype (Figure 3A and D), consistent with a previous report [42], yet dynole 34-2 did not affect bipolar spindle formation (Figure 3A and B) or centriole cohesion (Figure 3A and D). Therefore clathrin TD inhibition via pitstop 2 causes multipolar spindles due to a loss of centriole cohesion, indicating that clathrin has an additional metaphase function in maintaining spindle pole integrity.

### Pitstop does not impair clathrin-dependent mitotic spindle regrowth

**Figure 4:**
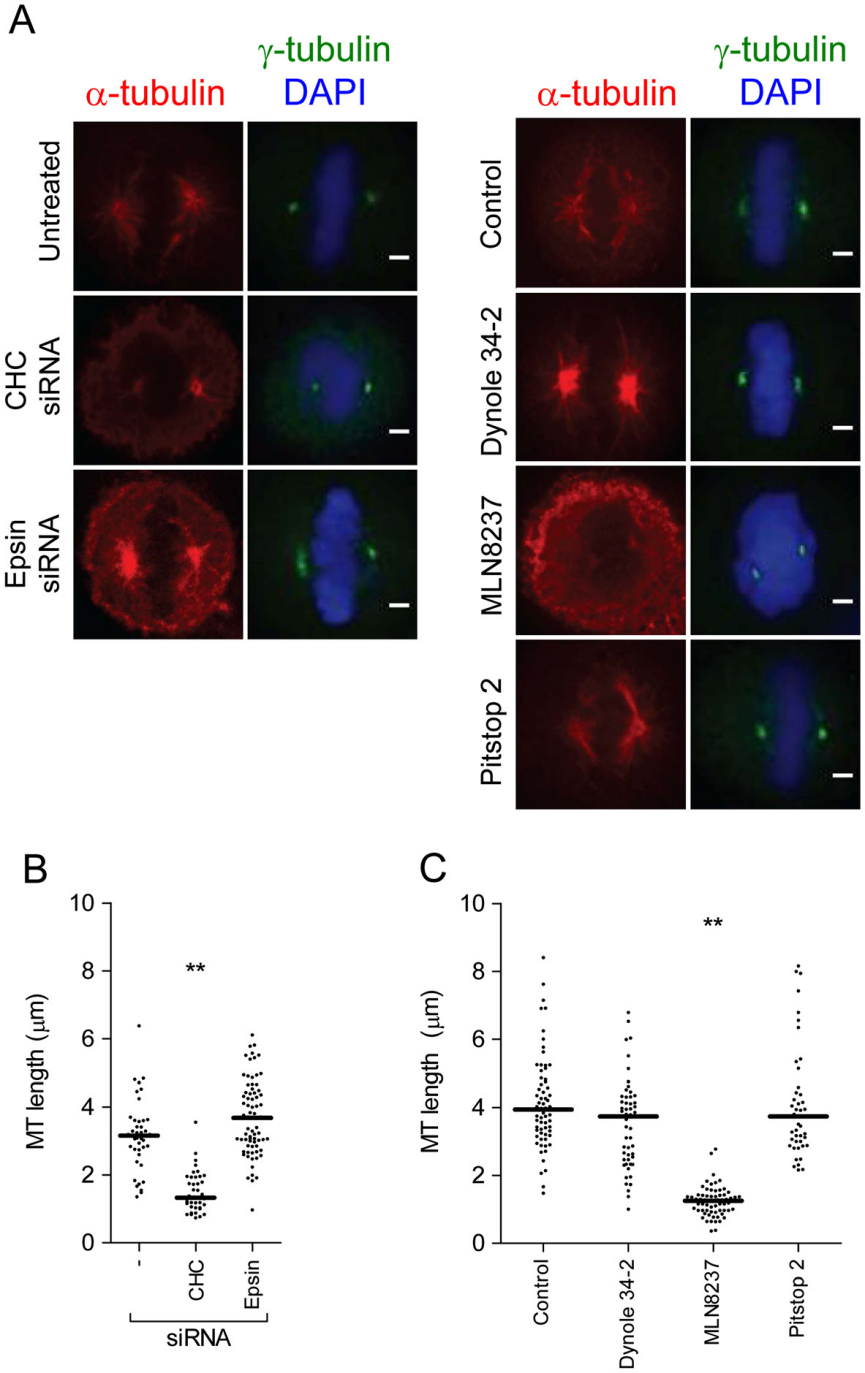
**Pitstop 2 does not affect microtubule nucleation from the mitotic spindle poles. A,** Metaphase synchronized HeLa cells were treated as described in Figure 2C then subjected to a MT regrowth assay whereby the MTs were allowed to regrow for 5 mins following depolymerization. Cells were fixed and stained for γ-tubulin (green), α-tubulin (red), and DNA (DAPI, blue). **B-C**, The dot blots show the length of the longest MT grown from each spindle pole in HeLa cells treated with the indicated siRNA (**B**) or drugs (**C**) as described in **A**. The median MT length in each experimental condition is indicated by the solid black line. n≥30 per sample. Statistical significance was determined by a Student’s *t*-test (* p < 0.05, ** p < 0.01).

Our next goal was to determine if the action of pitstop 2 at the spindle was mediated by its TD. A complex of phospho-TACC3 and ch-TOG is known to recruit clathrin to the mitotic spindle and that the trimeric complex is required for both assembly and stability of the spindle [27]. Phospho-TACC3 binds CHC via its linker domain and first CHC repeat and this interaction does not appear to require the TD of clathrin [13]. Therefore, the initial stage of spindle assembly requires CHC but may not require the function of its TD at this time point, and thus would be expected to be pitstop 2–resistant. We approached this by assessing the rapid spindle MT regrowth that occurs following cold-induced MT depolymerisation. After 1 min recovery of cells at 37°C the initial average spindle MT length of approximately 1 μM in metaphase cells was not different between all experimental conditions (untreated, vehicle-treated, pitstop 2, MLN8237, dynole 34–2, CHC and epsin siRNA) (Additional file 5: Figure S5A). However, in CHC-depleted cells and in cells treated with MLN8237 MT regrowth was significantly impaired after 5 min recovery (Figure 4A-C) and TACC3 spindle localization was severely disrupted (Additional file 6: Figure S6). In contrast, regrowth rates were unaffected in cells treated with pitstop 2, dynole 34–2 or epsin siRNA (Figure 4A-C) and the localization of CHC (Figure 1C) and TACC3 (Additional file 6: Figure S6) at the mitotic spindle was also unaffected. After 30 min regrowth, the length of the MTs in CHC-depleted cells and in cells treated with MLN8237 was comparable to control cells (Additional file 5: Figure S5B) indicating that reformation of the mitotic spindle is not abolished but slowed. MT regrowth from interphase centrosomes that generate non-mitotic spindle fibres was not disrupted by any experimental condition (Additional file 7: Figure S7). This is consistent with the lack of MT localization during interphase and a role for CHC and Aurora A kinase in regulating MTs during this stage of the cell cycle. Since the TD is not required for MT regrowth, these observations clearly support the above data that pitstop 2 action is most likely mediated by the TD. The data shows that the action of pitstop 2 on mitotic spindle integrity is occurring after the stage of clathrin recruitment specifically to spindle MTs by an as yet unknown key spindle protein.

### Pitstop 2 activates the spindle assembly checkpoint

**Figure 5:**
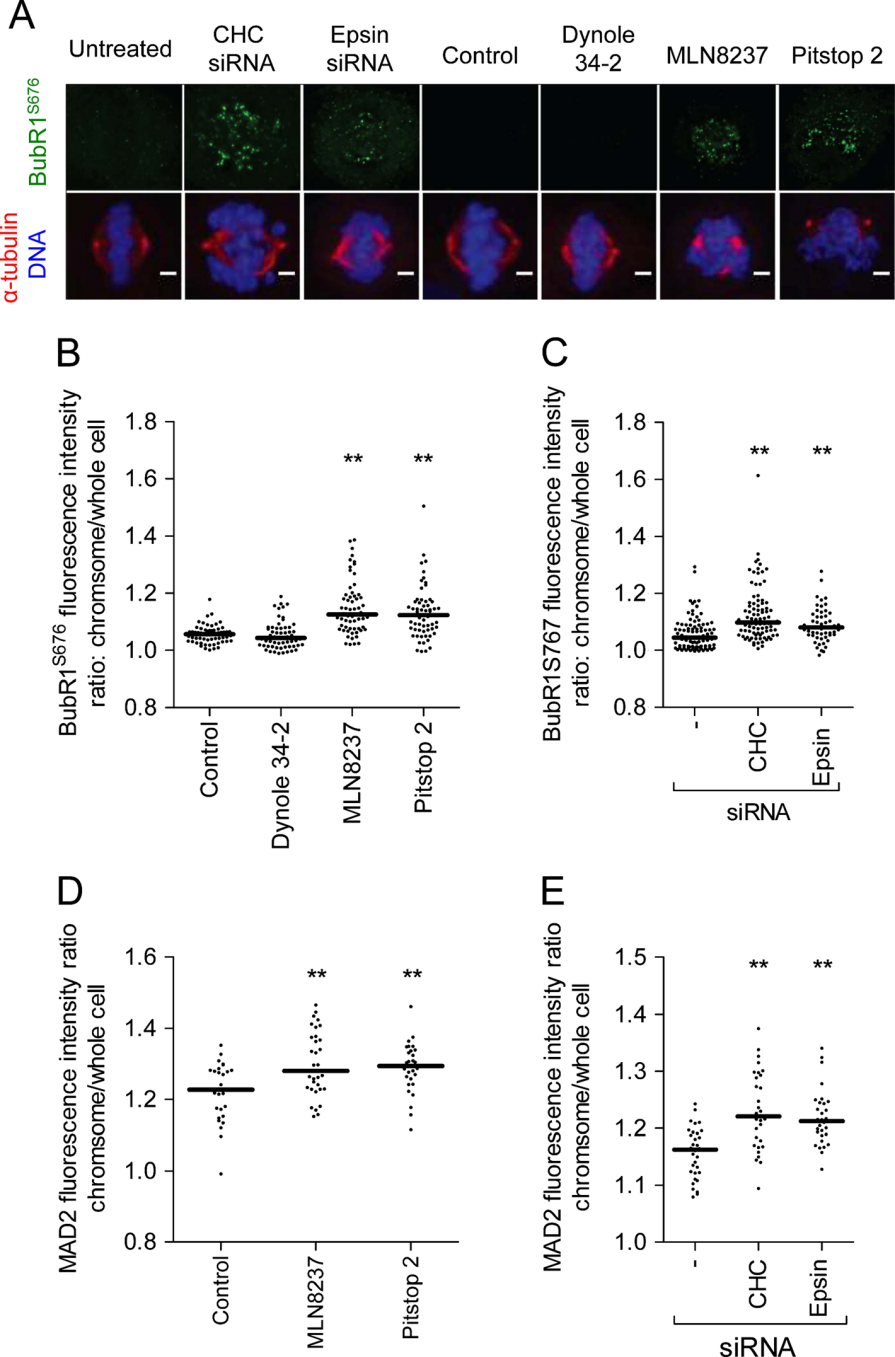
**Pitstop 2 activates the SAC. A**, Metaphase synchronized HeLa cells were treated as described in Figure legend 2C then fixed and stained for phospho-BubR1^S676^(green), α-tubulin (red) and DNA (DAPI, blue). Representative immunofluorescence microscopy images show that the amount of phospho-BubR1^S676^ on the chromosomes increases following treatment with pitstop 2, MLN8237, and following depletion of CHC or epsin. **B-C**, The dot blots show the fluorescence intensity ratio of BubR1^S676^ on the chromosomes/whole cell in individual HeLa cells treated with the indicated drugs (**B**) or siRNA (**C**). **D-E**, Metaphase synchronized HeLa cells were treated and processed as described in **A** except that they were stained for MAD2 instead of BubR1^S676^. The dot blots show the fluorescence intensity ratio of MAD2 on the chromosomes/whole cell in individual HeLa cells treated with the indicated drugs (**B**) or siRNA (**C**). The median fluorescence intensity ratio in all dots blots shown is indicated by the solid black line. n≥30 per sample from two independent experiments. Statistical significance was determined by a Student’s *t*-test (* p < 0.05, ** p < 0.01).

The SAC is activated when the K-fibres of the mitotic spindle have not attached correctly to the KTs that are associated with the centromeres of the chromosomes [2]. Since pitstop 2 disrupts K-fibres (Figure 2) we next asked whether pitstop 2 activates the SAC. BubR1 is a SAC protein that is phosphorylated on S676 and localizes to KTs upon SAC activation [34]. We quantified the fluorescence intensity of phospho-BubR1 at the spindle as a ratio of phospho-BubR1 on the chromosomes:whole cell. Pitstop 2, MLN8237, depletion of CHC or depletion of epsin all significantly increased the level of phospho-BubR1 at the spindle (Figure 5A-C). This indicates that the SAC is activated under these experimental conditions while dynole 34-2 did not activate the SAC. To independently confirm that pitstop 2 activates the SAC we quantified the level of MAD2 localisation to the chromosomes. MAD2 is only present on unattached KTs and is released once the checkpoint is satisfied [43,44]. The chromosomal localisation of MAD2 significantly increased following treatment with pitstop 2, MLN8237, CHC siRNA or epsin siRNA (Figure 5D and E). We conclude that pitstop 2 activates the SAC in an analogous manner to the Aurora A kinase inhibitor MLN8237.

### Pitstop 2 induces apoptosis and inhibits cell growth in dividing cancer cells

**Figure 6:**
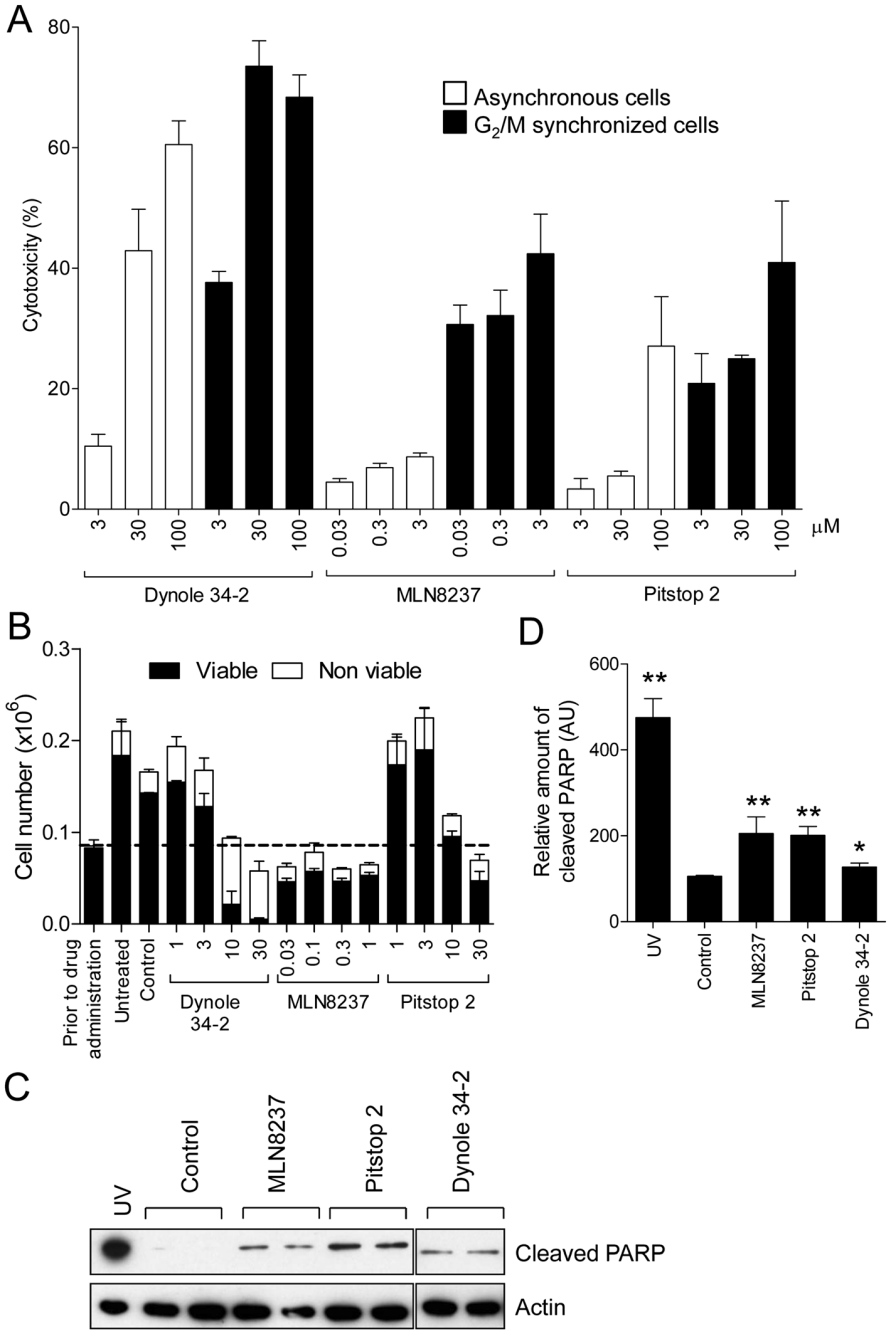
**Pitstop 2 causes cell death and inhibits cell growth in dividing cells. A**, Asynchronously growing and G_2_/M-synchronized HeLa cells were subject to an LDH assay following a 20 h treatment with increasing concentrations of pitstop 2, dynole 34-2 and MLN8237. The graph shows the amount of LDH in each experimental condition, as an indicator of cellular cytotoxicity, which was calculated as a percentage of total cell lysis. **B**, G_2_/M-synchronized HeLa cells were exposed to increasing concentrations of dynole 34-2, MLN8237 and pitstop 2 for 24 h. Total viable and non-viable cell number (mean ± S.D. from two independent experiments) were assessed by trypan blue. **C**, Cells were treated as described in **B**. Lysates were collected and immunoblotted for cleaved PARP. HeLa cells exposed to UV served as a positive control. Actin served as a loading control. **D**, Quantitative densitometric analysis of cleaved PARP levels shown in **C**. Graph (mean ± S.E.M. from five independent experiments) shows the relative amount of PARP in HeLa cells following treatment with the indicated conditions that have been normalised to background.

Mitotic inhibitors such as MLN8237 [5] which activate the SAC have anti-cancer properties due to their ability to inhibit cell proliferation and induce cell death following prolonged SAC activation [3,4]. We previously showed that pitstop 2 does not induce cell death in HeLa cells after an acute treatment for several hours [28]. However, those experiments were performed on asynchronously growing cells whereby only ~3% of cells are dividing at any given time and were specifically designed to show that the compound is not inherently cytotoxic. In contrast, we next set out to ask whether pitstop 2 will inhibit cell proliferation and induce cell death in mitotically dividing cells following a prolonged metaphase arrest, as occurs for other SAC activators. We first determined if pitstop 2 is cytotoxic and if this is selective for dividing cells by performing a lactate hydrogenase (LDH) release assay to assess membrane integrity, which is indicative of cell viability. After an acute 20 h treatment, dynole 34–2 is cytotoxic to cells in a dose-dependent manner, and toxicity increases significantly in dividing cells (Figure 6A), as previously shown [37]. Our previous report indicates that this cellular cytotoxicity occurs following cytokinesis failure. Specifically, dynole 34–2 caused cell death in G_2_/M synchronized HeLa cells at concentrations as low as 3 μM, but the equivalent induction of cell death was only evident at concentrations ≥30 μM in asynchronously growing cells (Figure 6A). Even after a 20 h exposure to pitstop 2, asynchronously growing HeLa cells were relatively resistant to this compound with <8% cell death at concentrations as high as 100 μM (Figure 6A), consistent with our previous report [28]. Pitstop 2 or MLN8237 exhibited analogous effects. G_2_/M synchronized HeLa cells were significantly more sensitive to either agent, with >21% (pitstop 2) of these cells undergoing cell death at concentrations as low as 3 μM (Figure 6A).

We next sought to determine if pitstop 2 also possessed anti-proliferative properties by scoring the total viable cell number using a trypan blue exclusion assay. G_2_/M synchronized HeLa cells were treated for 24 h immediately following release from the G_2_/M block. The total number of viable HeLa cells following pitstop 2, MLN8237 or dynole 34–2 treatment was markedly reduced in a dose-dependent manner (Figure 6B). At high concentrations of pitstop 2 (30 μM) the total viable cell number was less than the number of cells scored immediately prior to administration of the compound (dashed line). This indicates that pitstop 2 not only inhibits cell proliferation but also causes cell death. This was also evident following MLN8237 and dynole 34–2 treatment (Figure 6B). Taken together this indicates that pitstop 2 blocks proliferation and causes a much greater rate of cell death in dividing cells than non-dividing cells.

**Figure 7:**
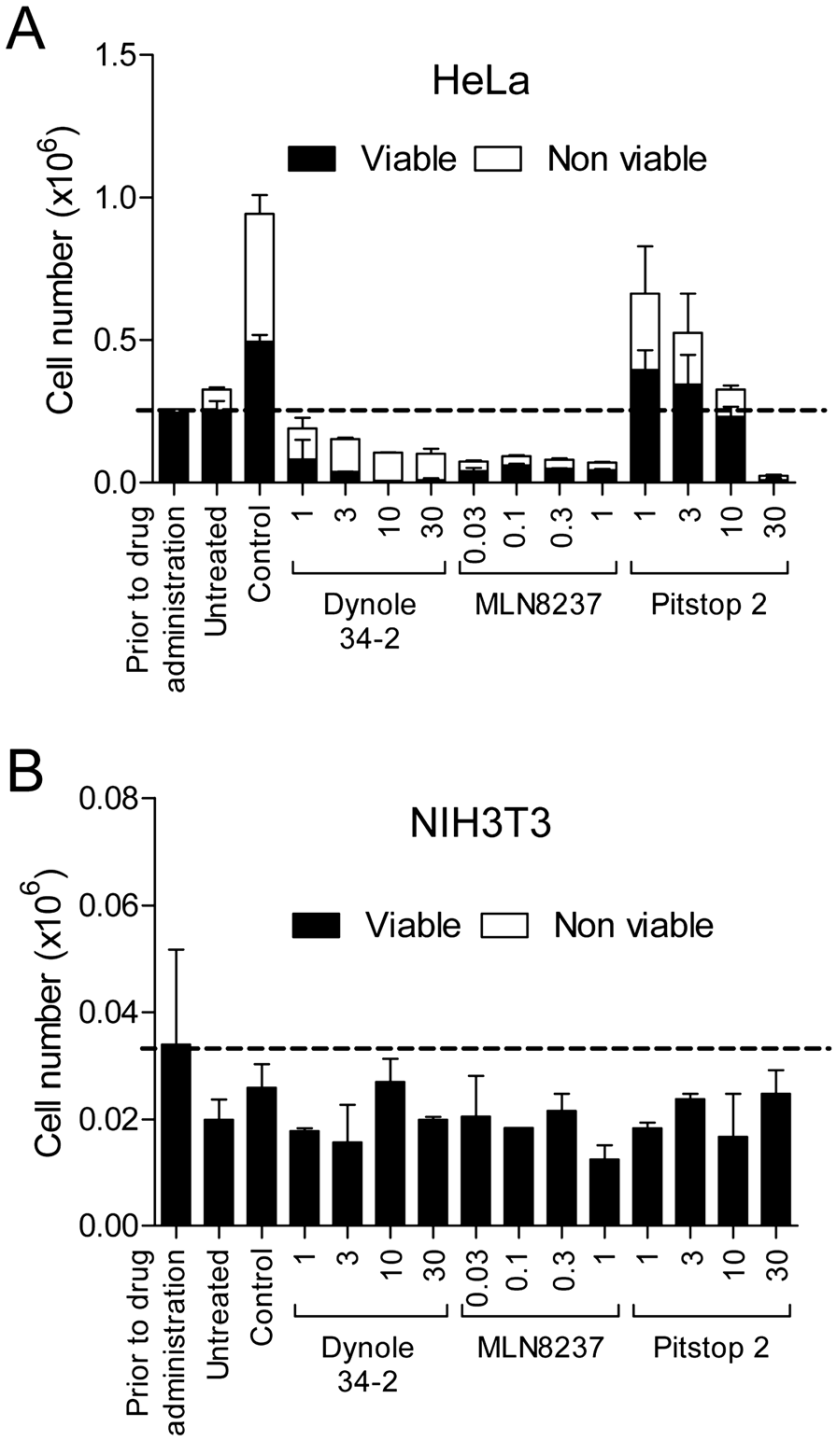
**Pitstop 2 does not affect the growth and viability of non-tumourigenic NIH3T3 fibroblasts.A-B**, HeLa **(A)** and NIH3T3 **(B)** cells were synchronized in mitosis and then treated with increasing concentrations of the indicated compounds at 24 h intervals. After 48 h, total viable and non-viable cell number (mean ± S.D. from two independent experiments) was assessed by trypan blue. Drug concentrations are indicated in μM units.

Pitstop 2-induced cell death was due to apoptosis since it was accompanied by an increase in PARP cleavage following a 24 h treatment (Figure 6C and D). PARP was also cleaved in cells treated with MLN8237, dynole 34-2 or following exposure to ultraviolet irradiation (UV; Figure 6C and D). Finally, we compared the growth of HeLa cells with non-tumourigenic fibroblasts, NIH3T3. While pitstop 2 blocked proliferation and induced cell death in G_2_/M-synchronized HeLa cells after 48 h (whereby cells were treated every 24 h, Figure 7A), the compound had no significant effect in NIH3T3 cells (Figure 7B). Collectively, these findings indicate that pitstop 2 has anti-proliferative and cytotoxic properties that appear to be relatively selective for dividing cancer cells. Thus, pitstop 2 is a new member of anti-mitotic compounds that causes mitotic arrest and SAC activation, with clathrin being the novel target.

## Discussion

Clathrin plays a non-endocytic function during mitosis that is downstream of the Aurora A kinase [38]. Inhibitors of Aurora A are currently being assessed in human clinical trials of cancer with promising success [5]. Here, we used the first clathrin inhibitor, pitstop 2, to illustrate that clathrin is also a valid target for the development of inhibitors that (i) are useful molecular tools to assess clathrin function and (ii) have the potential to be anti-cancer agents. Pitstop 2 induced mitotic phenotypes consistent with inhibition of clathrin, which included an increase in the mitotic index and width of the metaphase plate, loss of K-fibres and mitotic spindle integrity, chromosome mis-alignment and activation of the SAC. In an analogous manner to the Aurora A inhibitor, MLN8237, pitstop 2 inhibited cell proliferation and induced cell death exclusively in dividing cancer cells. Non-tumourigenic fibroblasts were not affected by this compound. Thus, our findings suggest that the clathrin TD inhibitor, pitstop 2, possesses anti-mitotic and anti-cancer properties consistent with other SAC activating compounds.

We confirm the mitotic role of clathrin in maintaining integrity of the mitotic spindle by stabilizing K-fibres. However, we reveal an additional mitotic role for clathrin in maintaining spindle pole integrity by participating in centriole cohesion. Depletion of CHC and pitstop 2 treatments caused an increase in the number of multipolar spindles whereby the spindle poles frequently contained only one centriole. Cyclin G-associated kinase (GAK) is a binding partner of CHC and required for CME [45]. Like CHC, it has recently been reported to also function during metaphase in an endocytic-independent manner for correct chromosome segregation. Like CHC, GAK localizes to the mitotic spindle and GAK-depleted cells phenocopy the mitotic defects induced by depletion of CHC [46]. One of the mitotic phenotypes observed in GAK-depleted cells is the presence of multipolar spindles that contain only one centriole at the spindle pole. It was proposed that this phenotype was due to microtubule forces, as mis-aligned chromosomes would generate pushing and pulling forces on the mitotic spindle resulting in fragmentation of the spindle poles. CHC is lost from the mitotic spindle in GAK-depleted cells [46] and thus a similar hypothesis could also explain the loss of centriole cohesion observed in CHC-depleted and pitstop 2–treated cells. Therefore CHC appears to play an indirect role in maintaining spindle pole integrity and centriole cohesion via its role in bridging K-fibres for correct chromosome congression and segregation.

The mitotic spindle localization of clathrin was not perturbed by pitstop 2. This was surprising given that pitstop 2 binds the TD of CHC [28], which is within the N-terminal region of clathrin known to be required for its mitotic spindle localization [11,21]. The crystal structure of the CHC-TD (residues 1–364) reveals a classical seven-bladed β-propeller that contains a clathrin-box LϕXϕ [DE] (where ϕ is a bulky hydrophobic amino acid, X is any and brackets enclose alternatives) and W-box motifs for association with binding partners [47,48], however some proteins may bind to other blades of the TD such that there may be up to 4 separate protein interaction sites in the TD [49]. Pitstop 2 docks into the cleft comprising the clathrin-box between blades 1 and 2 of the β-propellor [28]. Clathrin does not bind MTs directly, suggesting that clathrin is tethered to the mitotic spindle via other interactions that may involve other parts of clathrin such as the W-box. In support of this idea, phospho-TACC3 binds CHC via its linker domain and first CHC repeat and does not appear to require the TD of clathrin [13]. B-Myb [50] and GAK [46] have been implicated in recruiting clathrin to the mitotic spindle and thus represent potential W-box binding partners during mitosis. Clathrin is thought to recruit TACC3 to the mitotic spindle via interactions that do not dependent on the TD [13]. Consistent with this idea, we show that in contrast to depletion of CHC, pitstop 2 does not affect TACC3 recruitment to the mitotic spindle. Nor does it impair microtubule regrowth and initiation of mitotic spindle formation, which is in contrast to that caused by depletion of CHC. Thus, pitstop 2 does not induce an aberrant mitotic phenotype by blocking recruitment of clathrin to the mitotic spindle but rather appears to inhibit clathrin function once it is stationed at the spindle.

Anti-mitotic compounds that activate the SAC, such as MLN8237, are being developed as a new class of anti-cancer agents due to their ability to prolong metaphase arrest and subsequently inhibit cell proliferation and induce cell death in dividing cancer cells [3,4]. As a result, many of these targeted inhibitors reduce tumour volume *in vivo*[3] and are being assessed in cancer clinical trials [5]. We show that pitstop 2 possesses anti-cancer properties since it phenocopies SAC activating compounds like MLN8237 or CHC knockdown. Aurora A mediates its function through many other proteins in addition to clathrin and therefore Aurora A inhibitors induce a plethora of phenotypes [51]. This could potentially result in unwanted side effects and reduced efficacy for cancer patients. Therefore, we predict that clathrin TD inhibitors may be more targeted, raising the opportunity for them to be potentially more efficacious compounds for the treatment of cancer. Pitstop 2 was not as potent as MLN8237 at inducing aberrant mitotic phenotypes, inhibiting cell proliferation and inducing cell death, and new generation analogues are required. Our findings provide proof-of-concept that clathrin is a valid target for the development of small molecule inhibitors that can be exploited as new strategies to design anti-cancer therapeutics. Pitstop 2 therefore represents a new lead compound amenable to drug development.

## Competing interests

The authors declare no competing interest.

## Authors’ contributions

CMS designed and conducted experiments as well as carried out data analysis. VH, AM and PJR developed clathrin inhibitors, participated in intellectual discussion of the data and manuscript writing. MC contributed to experimental design, co-ordination of the project, data analysis and manuscript writing. All authors read and approved the manuscript.

## Supplementary Material

Additional file 1 Figure S1 Structures of compounds. The chemical structures of pitstop 2 (A), MLN8237 (B) and dynole 34-2 (C). Click here for file

Additional file 2 Figure S2 Depletion of CHC or epsin. Cells were depleted of the indicated protein by siRNA for 72 hours. Lysates were collected and immunoblotted for CHC, epsin and γ-tubulin. CHC and epsin siRNAs caused a >90% depletion of the target protein. Click here for file

Additional file 3 Figure S3 Schematic illustration of the time-line of experimental procedures. Click here for file

Additional file 4 Figure S4 Effect of pitstops on endocytosis. A quantitative high-throughput endocytosis assay using Texas Red-Tfn uptake in HeLa cells pre-treated with increasing concentrations of pitstop 2 and dynole 34-2 for 30 min. Data are expressed as mean ± 95% confidence intervals (CI) for triplicates and ~1200 cells. Similar results were obtained in three independent experiments. Click here for file

Additional file 5 Figure S5 Microtubule regrowth in mitotic cells. A-B, Metaphase-synchronized HeLa cells were treated with the indicated drugs (left) or siRNA (right) then subjected to a MT regrowth assay after 30 min cold exposure, whereby the MTs were allowed to regrow at 37°C for 1 min (A) and 30 min (B) following depolymerization. Cells were fixed and stained for γ-tubulin (green), α-tubulin (red), and DNA (DAPI, blue). The dot blots show the length of the longest MT grown from each spindle pole in HeLa cells treated with the indicated drugs (left) or siRNA (right). The median MT length in each experimental condition is indicated by the solid black line. n ≥ 30 per sample. Statistical significance was determined by a Student’s *t*-test (* p < 0.05, ** p < 0.01). Click here for file

Additional file 6 Figure S6 TACC3 recruitment is not affected by pitstop compounds. A, Metaphase synchronized HeLa cells treated with the indicated siRNA or drugs were stained for TACC3 (green), α-tubulin (red), and DNA (DAPI, blue). Representative immunofluorescence microscopy images illustrate that that MLN8237 and CHC siRNA treatment decreases TACC3 recruitment to the mitotic spindle. B-C, Quantitation of data described in A. The dot blots show the fluorescence intensity ratio of TACC3 on the mitotic spindle/whole cell in individual HeLa cells treated with the indicated drugs (B) or siRNA (C). The median fluorescence intensity ratio in all dots blots shown is indicated by the solid black line. n ≥ 30 per sample. Statistical significance was determined using a Student’s *t*-test (* p < 0.05, ** p < 0.01). Click here for file

Additional file 7 Figure S7 Effect of pitstop 2 on microtubule regrowth in interphase cells. A-B, Asynchronously growing HeLa cells were treated with the indicated compounds (left) and siRNA (right) then subjected to a MT regrowth assay whereby the MTs were allowed to regrow for 1 min (A) and 5 min (B) at 37°C following a cold depolymerization. Cells were fixed and stained for γ-tubulin (green), α-tubulin (red), and DNA (DAPI, blue). The dot blots show the length of the longest MT grown from each centrosome in HeLa cells in interphase treated with the indicated drugs (left) or siRNA (right). The median MT length in each experimental condition is indicated by the solid black line. n ≥ 30 per sample. Statistical significance was determined by a Student’s *t*-test (* p < 0.05, ** p < 0.01). Click here for file
