# Supplementary figures and images for: Inhibition of clathrin by pitstop 2 activates the spindle assembly checkpoint and induces cell death in dividing HeLa cancer cells
Source: Mol Cancer. 2013 Jan 17;12:4. doi: 10.1186/1476-4598-12-4 (PMC3567983; doi:10.1186/1476-4598-12-4)

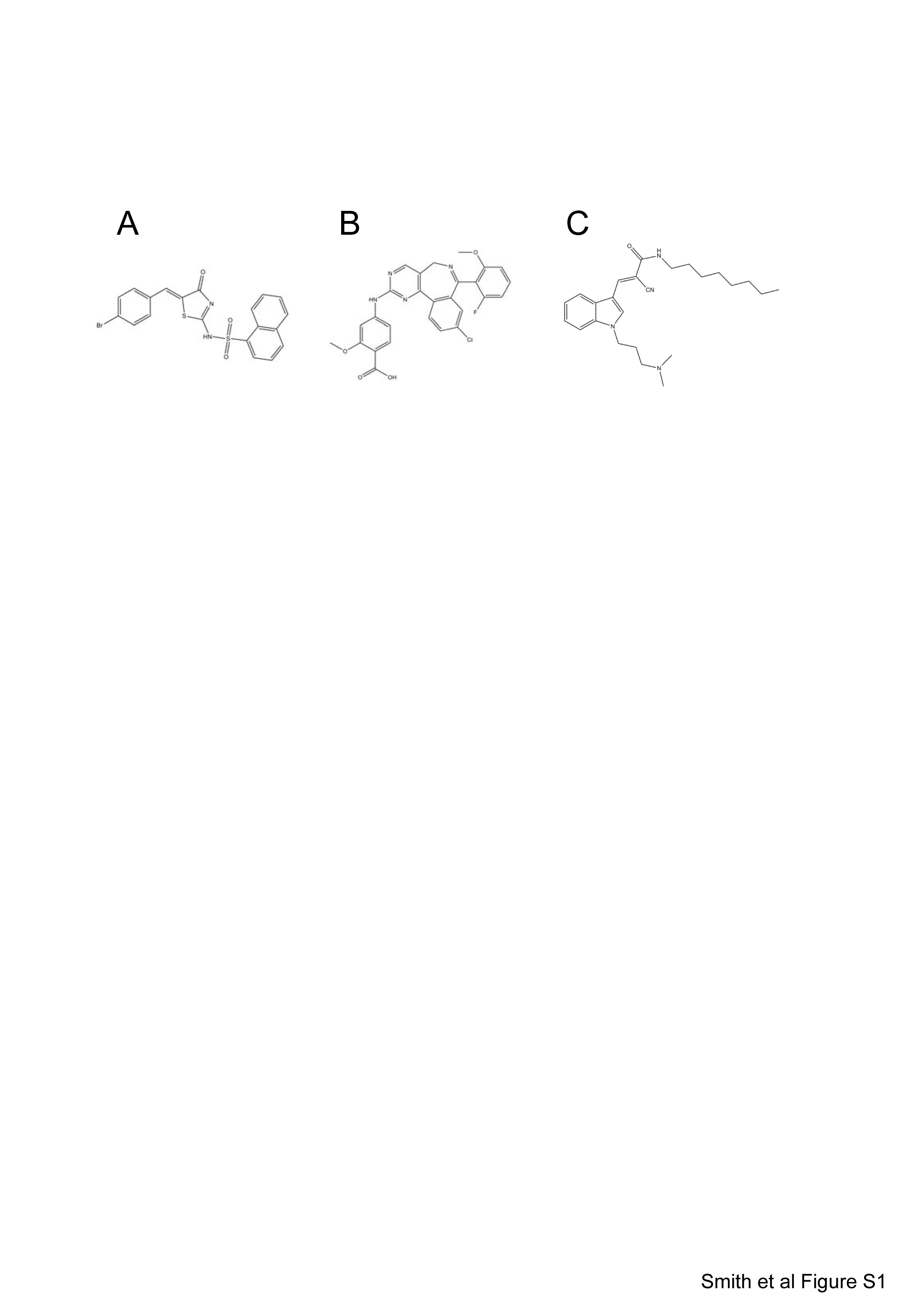

Supplement: Additional file 1 Figure S1 — Structures of compounds. The chemical structures of pitstop 2 (A), MLN8237 (B) and dynole 34-2 (C). [file 1476-4598-12-4-S1.tiff]

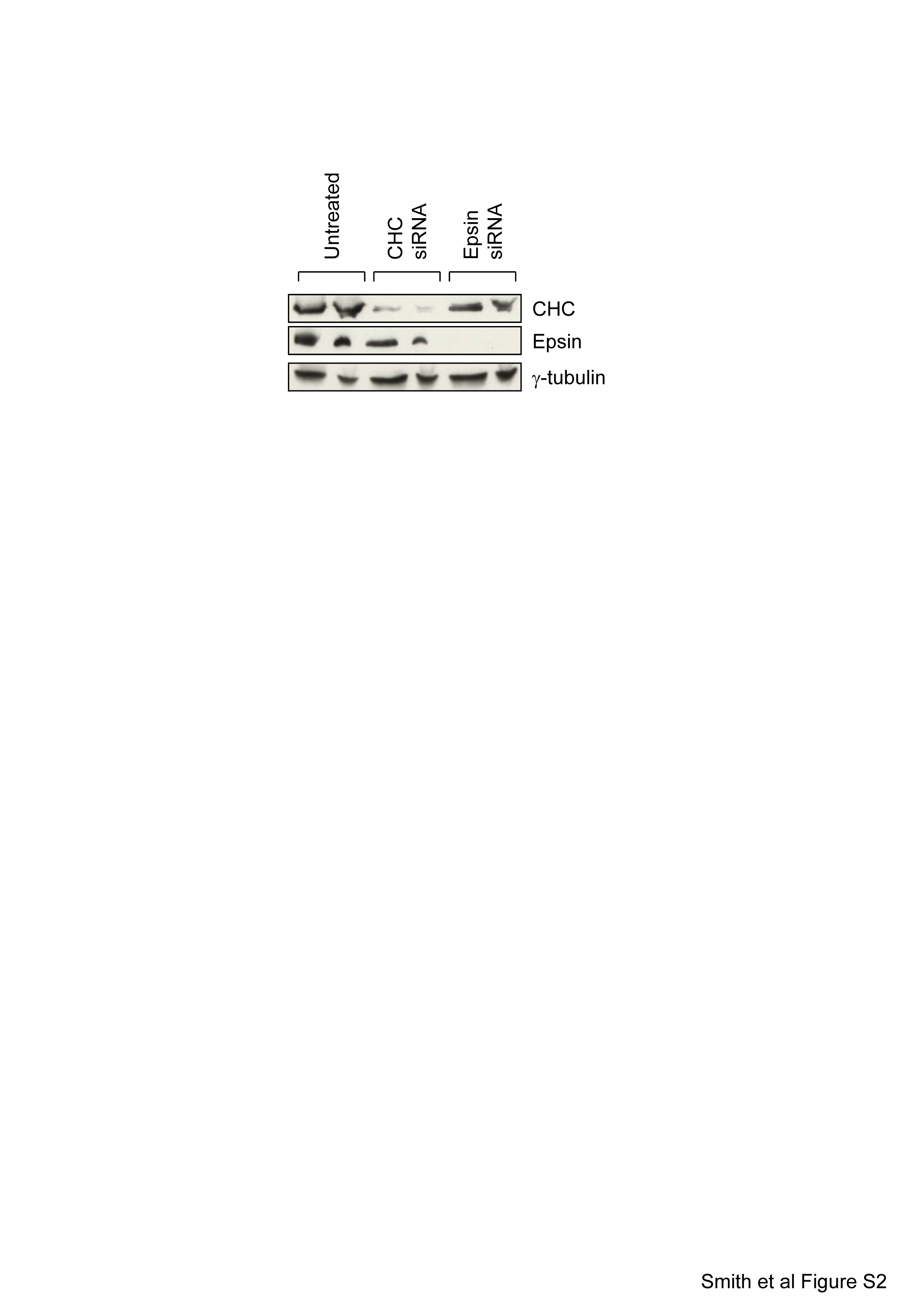

Supplement: Additional file 2 Figure S2 — Depletion of CHC or epsin. Cells were depleted of the indicated protein by siRNA for 72 hours. Lysates were collected and immunoblotted for CHC, epsin and γ-tubulin. CHC and epsin siRNAs caused a >90% depletion of the target protein. [file 1476-4598-12-4-S2.tiff]

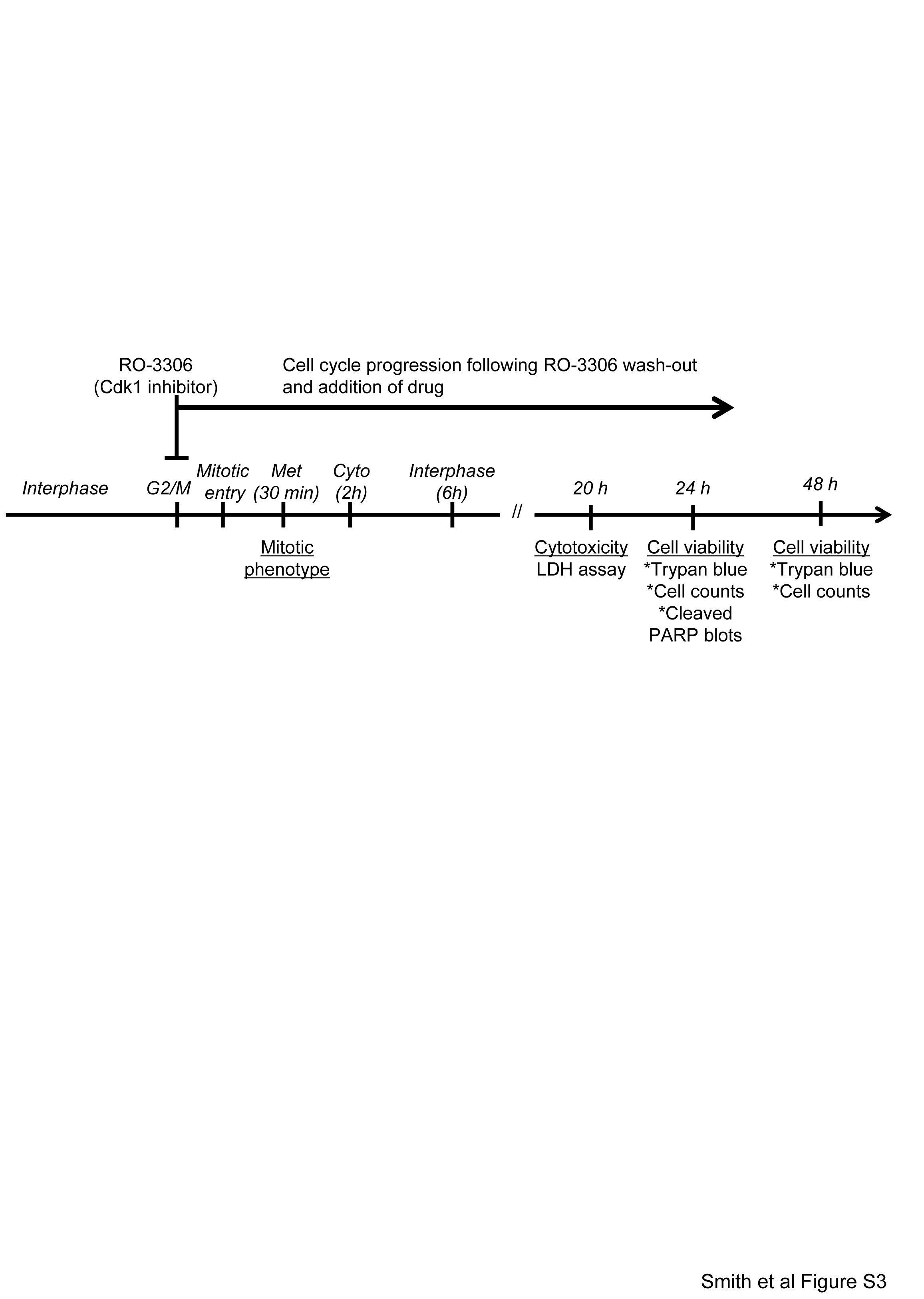

Supplement: Additional file 3 Figure S3 — Schematic illustration of the time-line of experimental procedures. [file 1476-4598-12-4-S3.tiff]

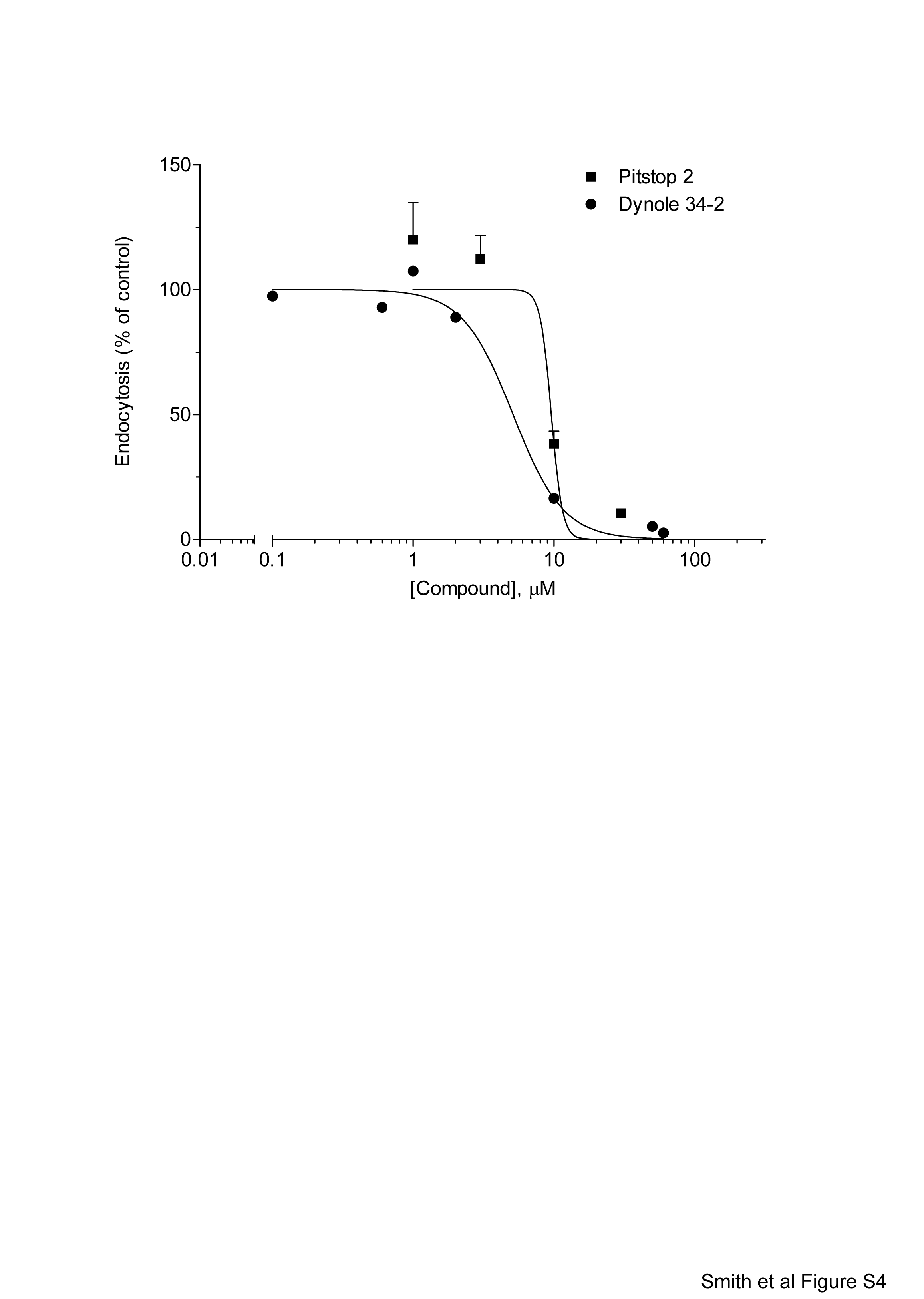

Supplement: Additional file 4 Figure S4 — Effect of pitstops on endocytosis. A quantitative high-throughput endocytosis assay using Texas Red-Tfn uptake in HeLa cells pre-treated with increasing concentrations of pitstop 2 and dynole 34-2 for 30 min. Data are expressed as mean ± 95% confidence intervals (CI) for triplicates and ~1200 cells. Similar results were obtained in three independent experiments. [file 1476-4598-12-4-S4.tiff]

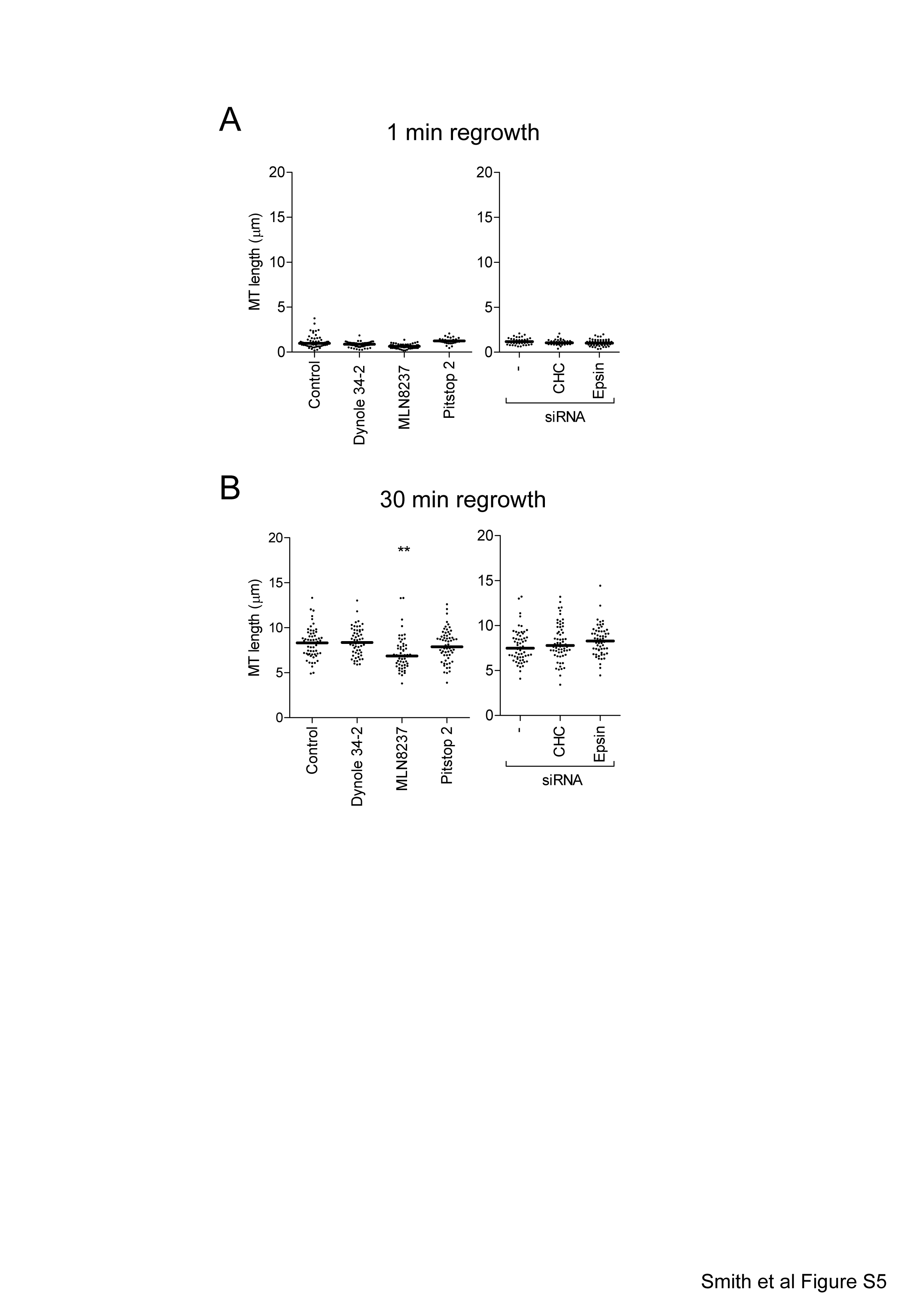

Supplement: Additional file 5 Figure S5 — Microtubule regrowth in mitotic cells. A-B, Metaphase-synchronized HeLa cells were treated with the indicated drugs (left) or siRNA (right) then subjected to a MT regrowth assay after 30 min cold exposure, whereby the MTs were allowed to regrow at 37°C for 1 min (A) and 30 min (B) following depolymerization. Cells were fixed and stained for γ-tubulin (green), α-tubulin (red), and DNA (DAPI, blue). The dot blots show the length of the longest MT grown from each spindle pole in HeLa cells treated with the indicated drugs (left) or siRNA (right). The median MT length in each experimental condition is indicated by the solid black line. n ≥ 30 per sample. Statistical significance was determined by a Student’s t-test (* p < 0.05, ** p < 0.01). [file 1476-4598-12-4-S5.tiff]

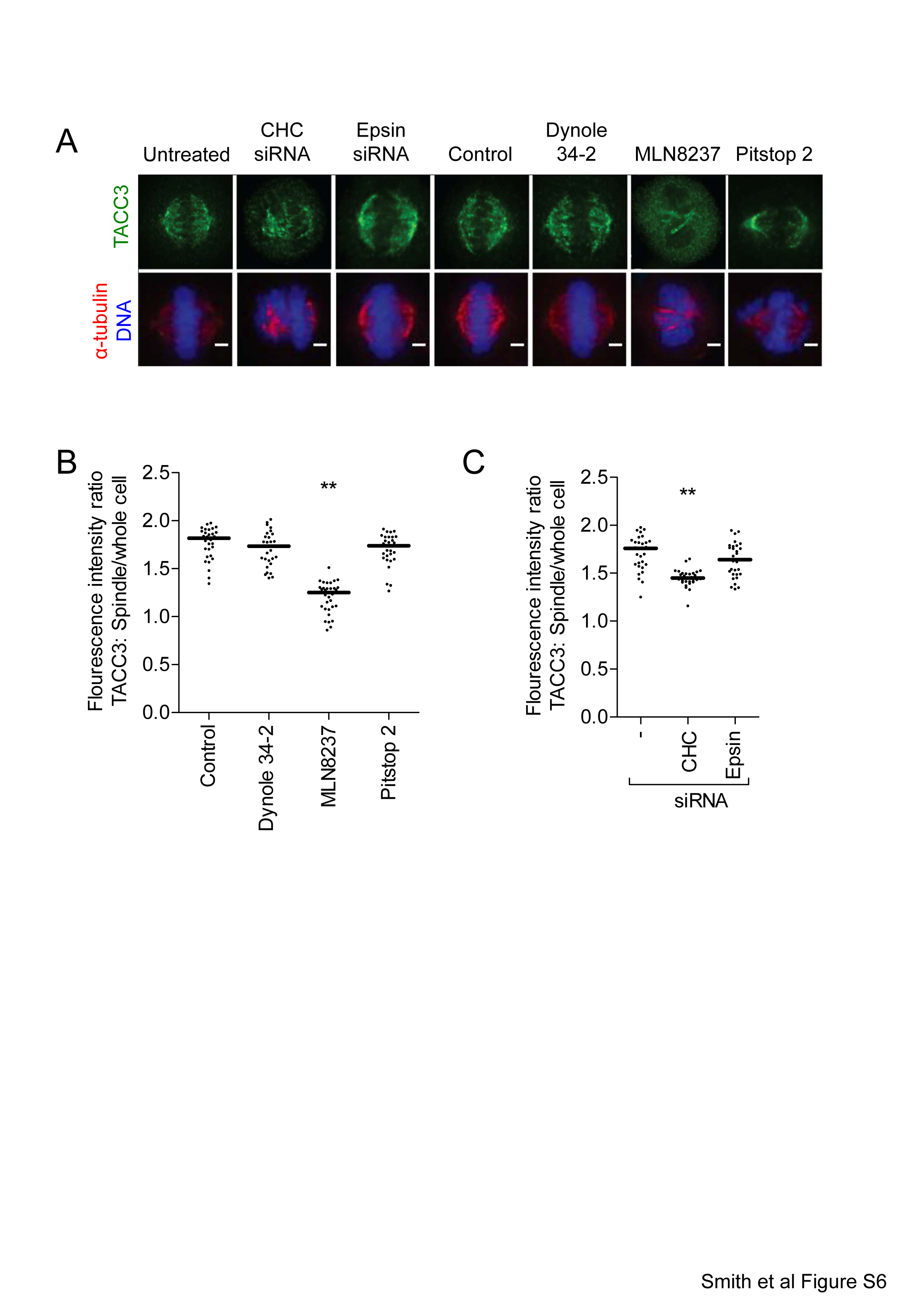

Supplement: Additional file 6 Figure S6 — TACC3 recruitment is not affected by pitstop compounds. A, Metaphase synchronized HeLa cells treated with the indicated siRNA or drugs were stained for TACC3 (green), α-tubulin (red), and DNA (DAPI, blue). Representative immunofluorescence microscopy images illustrate that that MLN8237 and CHC siRNA treatment decreases TACC3 recruitment to the mitotic spindle. B-C, Quantitation of data described in A. The dot blots show the fluorescence intensity ratio of TACC3 on the mitotic spindle/whole cell in individual HeLa cells treated with the indicated drugs (B) or siRNA (C). The median fluorescence intensity ratio in all dots blots shown is indicated by the solid black line. n ≥ 30 per sample. Statistical significance was determined using a Student’s t-test (* p < 0.05, ** p < 0.01). [file 1476-4598-12-4-S6.tiff]

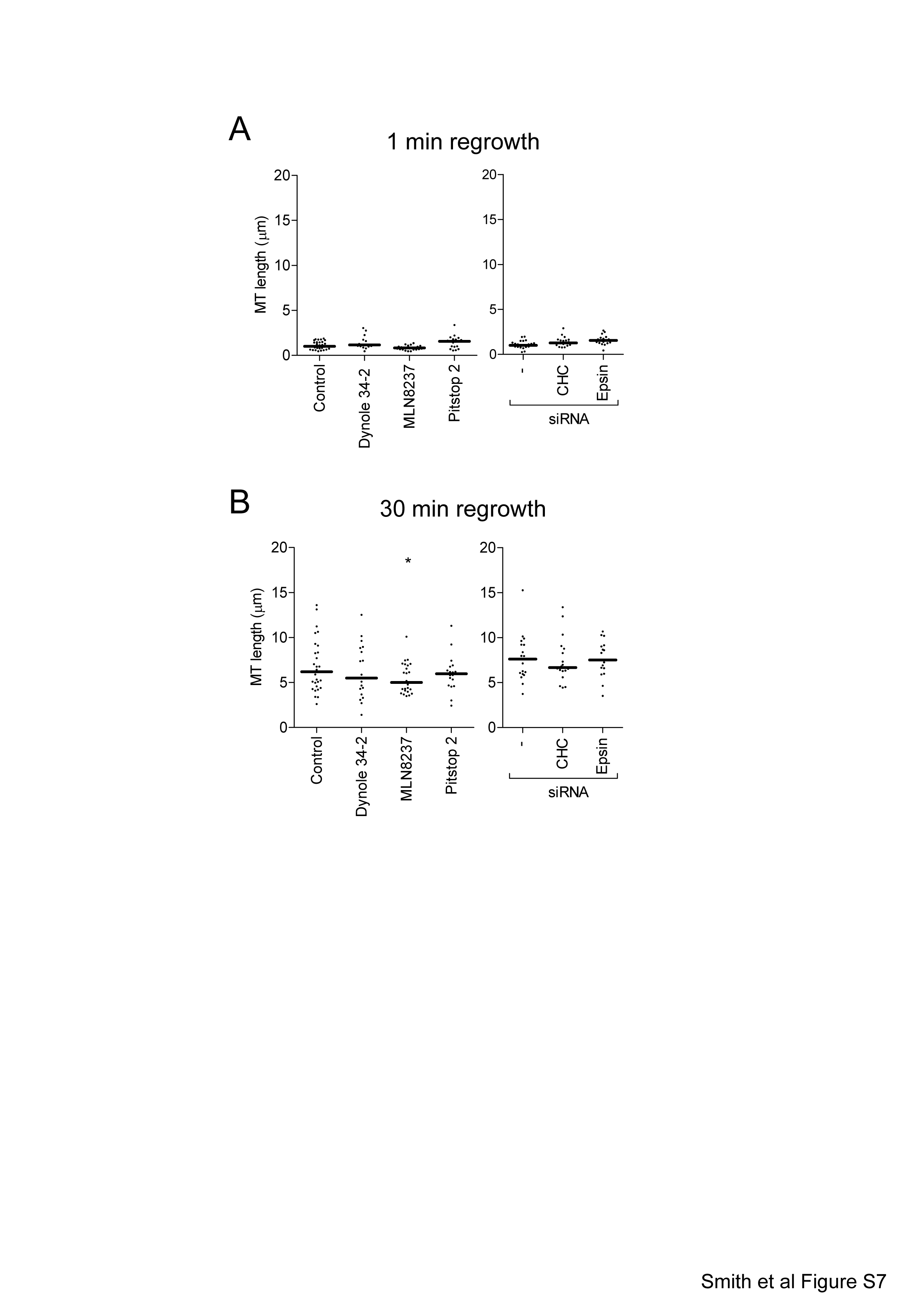

Supplement: Additional file 7 Figure S7 — Effect of pitstop 2 on microtubule regrowth in interphase cells. A-B, Asynchronously growing HeLa cells were treated with the indicated compounds (left) and siRNA (right) then subjected to a MT regrowth assay whereby the MTs were allowed to regrow for 1 min (A) and 5 min (B) at 37°C following a cold depolymerization. Cells were fixed and stained for γ-tubulin (green), α-tubulin (red), and DNA (DAPI, blue). The dot blots show the length of the longest MT grown from each centrosome in HeLa cells in interphase treated with the indicated drugs (left) or siRNA (right). The median MT length in each experimental condition is indicated by the solid black line. n ≥ 30 per sample. Statistical significance was determined by a Student’s t-test (* p < 0.05, ** p < 0.01). [file 1476-4598-12-4-S7.tiff]
